# Temporalis Muscle Thickness as a Prognostic Factor for 30-Day, 90-Day, and Overall Mortality in Newly Diagnosed Glioblastoma

**DOI:** 10.7759/cureus.103895

**Published:** 2026-02-19

**Authors:** Mohamed Abouelleil, Omar Nabulsi, Sabah Hamidi, Ankush Chandra, Lara Massie, Tarek Mansour, Momina Mustaquim, Mohamed Macki, Tobias Walbert, Adam Robin, Brent Griffith, Bilaluddin Jamaluddin, Victor Chang, Steven N Kalkanis, Ian Y Lee, Hesham M Zakaria

**Affiliations:** 1 Neurological Surgery, Spectrum Health Medical Group, Grand Rapids, USA; 2 School of Medicine, Rush University Medical Center, Chicago, USA; 3 Medicine, Rush University Medical Center, Chicago, USA; 4 Neurological Surgery, University of Texas Health Science Center at Houston, Houston, USA; 5 Neurosurgery, Allegheny Health Network, Pittsburgh, USA; 6 Neurological Surgery, Henry Ford Health System, Detroit, USA; 7 Neurology, Henry Ford Health System, Detroit, USA; 8 Radiology, Henry Ford Health System, Detroit, USA; 9 Cardiac Intensive Care Unit, Lenox Hill Hospital, New York City, USA; 10 Neurological Surgery, Henry Ford Health System, West Bloomfield, USA

**Keywords:** frailty, glioblastoma, sarcopenia, survival, temporalis muscle

## Abstract

Background: Glioblastoma (GBM) is the most common primary malignant brain tumor in adults. Accurate prognostic biomarkers are needed to guide care and treatment pathways across the spectrum of age. Temporal muscle thickness (TMT) is an accessible parameter that has been recognized as a prognostic marker for GBM. In addition, frailty, as measured by sarcopenia, has been proven to predict overall survival in other oncologic processes.

Objective: We evaluated whether sarcopenia, as measured by temporalis muscle thickness, has prognostic value for predicting survival in GBM. We aimed to confirm its prognostic accuracy and compare it to other survival markers.

Methods: A prospective GBM database identified 257 patients undergoing initial diagnostic surgery at Henry Ford Hospital in Detroit, Michigan. Sarcopenia was quantified by temporalis muscle thickness and grouped into tertiles. Mortality hazard ratios were calculated using multivariate analysis.

Results: After multivariate analysis, sarcopenia at the time of initial surgery was the only factor associated with mortality at 30 days postoperatively (OR 0.10, *P* = 0.030). Analysis demonstrated that mortality at 30 days had no association with gender, past medical history, tumor focality, tumor volume, tumor eloquence, or extent of resection. Sarcopenia at initial surgery predicted 90-day postoperative mortality; the most sarcopenic patients (first tertile) had greater mortality than those in the second (OR 0.28, *P *= 0.021) and third tertiles (OR 0.04, *P *= 0.003). Sarcopenia predicted overall mortality, greater in the first tertile than the second (OR 0.41, *P *< 0.001) and third tertiles (OR 0.41, *P *< 0.001). Sarcopenia compared favorably to other predictors of mortality, including initiation of postoperative temozolomide and radiation treatment (OR 0.27, *P* < 0.001), gross total resection (OR 0.54, *P *= 0.007), and O6-methylguanine-DNA methyltransferase (MGMT) methylation status (OR 0.44, *P* < 0.001). Kaplan-Meier survival curves represent differences in survival (log-rank p < 0.001).

Conclusions: Sarcopenia has prognostic value for predicting postoperative 30-day, 90­-day, and overall survival from diagnosis in GBM. The frailty/sarcopenia paradigm is independent of patient demographic, oncologic, genetic, surgical, and therapeutic factors. Temporalis muscle thickness assessment provides a simple method to help guide treatment decisions in affected adult populations.

## Introduction

Glioblastoma (GBM) is the most common and aggressive primary brain tumor. Despite refinement of surgical techniques and aggressive treatment with radiation and chemotherapy, median survival in GBM patients has not drastically improved over the last several years [1,2]. Beyond its oncologic burden, GBM also carries significant psychosocial consequences; recent evidence shows increased rates of suicidal ideation and attempts among brain tumor patients and survivors, underscoring the broader impact of this diagnosis and the importance of early risk stratification and awareness [3]. Establishing valid, accurate, and reliable markers of overall survival in patients with GBM prior to surgery would help stratify treatments for this at-risk population, ranging from aggressive to palliative. While there are several existing markers of survival in GBM, limitations prevent them from broad, universal application. The best available evidence suggests the extent of surgical resection is one of the strongest influencers of overall survival [4,5]; however, it is limited by dependence on surgeon’s technical skill, it can only be identified postoperatively, and not every patient is a candidate for extensive resection [6,7]. Tumor genotyping is predictive of tumor response to therapy; however, it requires tumor tissue and specialized resources limiting its global use [8,9]. Other patient-related factors that can be delineated preoperatively (tumor size, performance status, age/comorbidities, etc.) [10,11] may each contribute to survival, but they cannot individually guide the approach to GBM care.

Validated, comprehensive, and simple biomarkers of patient survival and fitness for treatment of GBM are needed. The ideal biomarker should be cost-effective, easily identifiable, and applied preoperatively. Its predictive capacity should be independent of other demographic, oncologic, genetic, surgical, and therapeutic factors. It should have a high validity and be practical enough that patients and clinicians use it for informed preoperative counseling and planning.

Frailty has the potential to be such a biomarker. While there is no comprehensive definition of patient frailty, it is generally understood as a decreased reserve to physiologic stressors and a common endpoint of senescence [12,13]. Clinically, patient frailty has been shown to predict morbidity and mortality after general surgery [14,15], and it has been shown to be strongly associated with decreased cancer patient survival [16]. However, frailty, with no standard definition, is onerous to quantify, and so sarcopenia (lack of muscle mass) is commonly used as its surrogate [17,18]. Sarcopenia has had success as a biomarker, being identified as an independent predictor of survival in various cancers, irrespective of surgical resection [19-25]. Sarcopenia is also an intuitive biomarker, as clinical experience reinforces the idea that in patients with identical cancers, one who is sarcopenic or cachectic will have a worse outcome than a more physically fit patient [17]. The frailty/sarcopenia paradigm is also a holistic approach to oncologic care, as it represents the patient’s overall health status and ability to tolerate treatment [17].

In prior studies, sarcopenia was defined by core muscle size and mostly identified on standard computed tomography (CT) or magnetic resonance imaging (MRI) [19-26]. Specifically, the average psoas muscle area was used to determine a patient’s sarcopenia status; patients with a small psoas muscle were considered sarcopenic, and patients with a large psoas were considered the antithesis. The use of the psoas muscle size as a hallmark for frailty is intuitive, as it is a core muscle vital for human ambulation and function. However, most patients with GBM do not undergo abdominal imaging, making it impractical to measure the psoas. Rather, another muscle identified on cranial imaging (frequent in GBM patients) can be utilized. Recent studies have confirmed that temporalis muscle thickness corresponds to psoas muscle area, and thus can also be used to quantify frailty/sarcopenia [27,28]. This finding that temporalis muscle thickness is associated with frailty and sarcopenia is also intuitive, as it is a muscle of mastication and will likely atrophy without usage or proper nutrition. Most conveniently and importantly, the temporalis muscle can be readily identified on perioperative MRI, which is done as a standard of care for patients with GBM.

A recent study conducted a systematic review to determine the prognostic implications of the temporalis muscle thickness in patients with GBM [29]. Therefore, due to its simplicity and universality, we elected to use temporalis muscle thickness as our marker of sarcopenia and sought to confirm whether sarcopenia can be used to independently predict survival from the time of initial diagnostic surgery in patients with newly diagnosed GBM. Additionally, we compared sarcopenia to other known biomarkers to predict overall mortality. 

Our study was previously posted to the Research Square preprint server on January 30, 2024.

## Materials and methods

Study design, setting, and participants

The study was conducted at Henry Ford Hospital in Detroit, Michigan, after obtaining approval from the Institutional Review Board (IRB #11482). Every patient whose data were used in this study provided consent. We used our prospectively collected cranial tumor bank database to identify consecutive patients with the diagnosis of GBM. Patients were included sequentially from 2014 through 2022, resulting in a final cohort of 257 adult patients, which provided sufficient power for analysis. Patients were excluded if their primary pathology was not GBM or if they had a previous history of lower-grade glioma.

Data sources, variables, and study size

**Figure 1:**
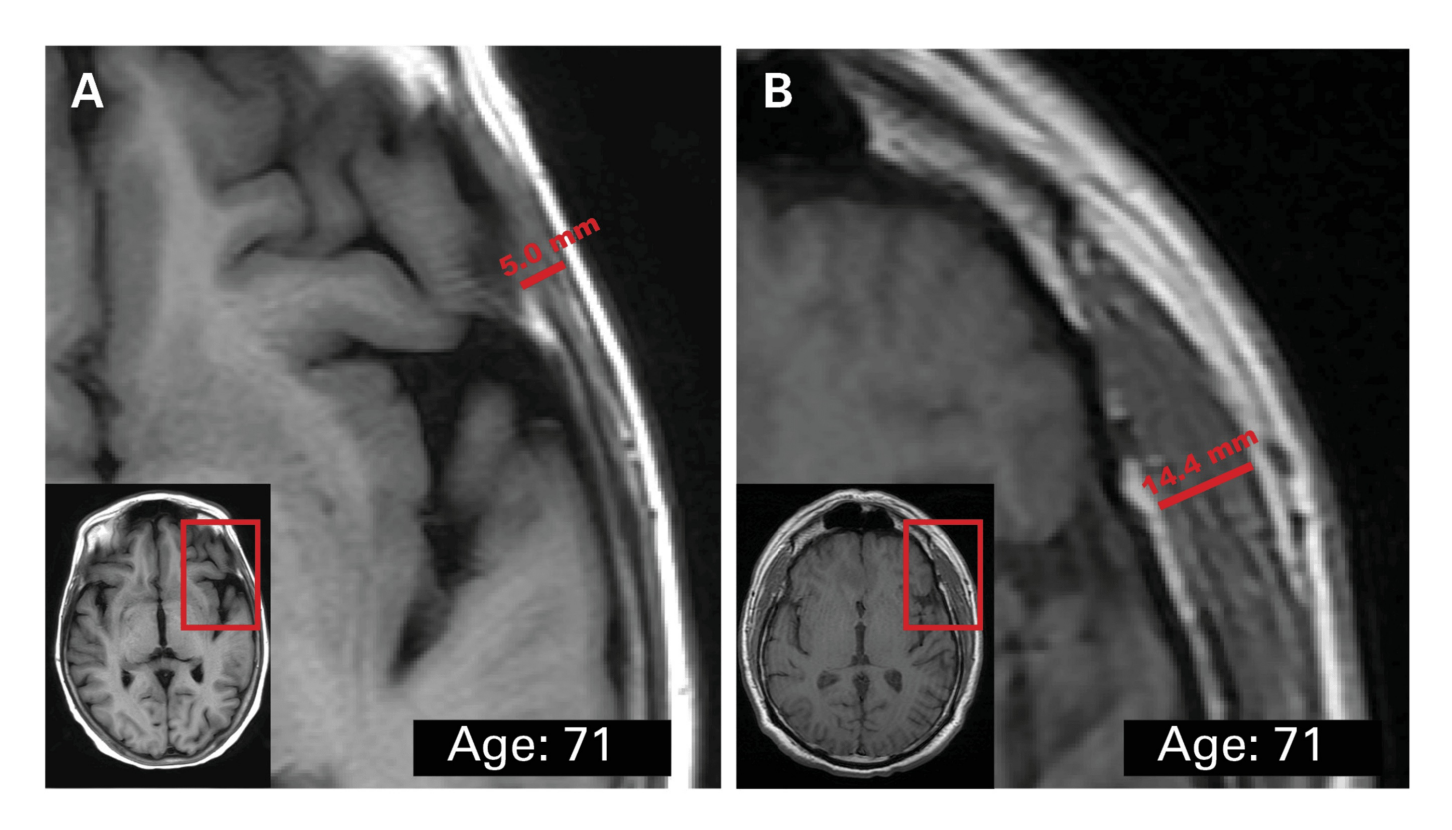
Perioperative measurement of temporalis muscle thickness on T1-weighted axial MRI at initial surgery for the diagnosis of GBM. Figure A depicts a frail 71-year-old male patient with a thin temporalis thickness of 5.0 mm. Figure B depicts a healthier 71-year-old male patient with a thick temporalis thickness of 14.4 mm.

Patient electronic medical records were retrospectively reviewed for data collection. We identified 257 adult patients (≥18 years) who underwent initial diagnostic surgery (biopsy or craniotomy for tumor resection) at our institution. Only patients with histopathologically confirmed GBM and available preoperative imaging adequate for temporalis muscle thickness measurement were included. Patients were excluded if they were younger than 18 years of age, had recurrent GBM, underwent prior cranial surgery or oncologic treatment before presentation at our institution, had incomplete electronic medical records, lacked required preoperative imaging, or did not have appropriate documented consent on file. Patients with conditions known to significantly alter skeletal muscle mass independent of cancer-related sarcopenia (e.g., neuromuscular disorders, advanced systemic inflammatory or cachectic diseases unrelated to GBM) were also excluded when documented. Temporalis thickness was measured at the time of the initial surgery for diagnosis of GBM using perioperative T1-weighted axial MRI (Fig. 1). Preoperative imaging was preferentially used, and T1-weighted MRI with contrast was used for those patients without non-contrast imaging. At our institution, postoperative MRI with contrast is performed on all patients undergoing craniotomy for GBM, and so postoperative imaging was used for measurements if preoperative imaging was inappropriate. Only preoperative imaging was used for patients with craniotomies that involved the temporalis. Temporalis thickness measurements were taken bilaterally along the long axis of the temporalis muscle, at the depth of the Sylvian fissure, and at the orbital roof by a single person (physician).

Tumor characteristics (volume, focality, laterality, eloquence, and location) were all measured from perioperative MRI. Tumor volume was measured with the ABC/2 method, using the borders of contrast enhancement on preoperative MRI [30]. Surgical site eloquence was defined as having contrast enhancement on perioperative MRI at the sensorimotor strip, dominant hemisphere perisylvian language areas, basal ganglia, internal capsule, thalamus, calcarine visual cortex, and periventricular visual fibers. Tumor location was defined as any contrast enhancement within a particular lobe or region (i.e., frontal, temporal, parietal, occipital, insular/deep nuclei, brainstem, and callosal), with most tumors encompassing multiple regions. The extent of resection was characterized via tumor bank notes; whenever there was ambiguity or missing data, it was verified by comparing pre-operative contrast enhancement with post-operative contrast enhancement. Gross total resection was compared against subtotal resection/biopsy. Post-operative chemotherapy and radiation were defined as the initiation of standard dosage of temozolomide and external beam radiation therapy (EBRT). Past medical history reviewed on electronic medical records included diabetes mellitus (DM), hypertension (HTN), seizure, hyperlipidemia (HLD), history of myocardial infarction (MI) or coronary artery disease (CAD), history of stroke or transient ischemic attack (TIA), and history of chronic obstructive pulmonary disease (COPD) or asthma. The primary outcome measure was mortality from the date of surgery.

Statistical methods

To analyze the association of temporalis size with survival, measurements of the temporalis muscle were divided into tertiles. The primary outcomes were 30-day mortality, 90-day mortality, and overall mortality (time to death). Survival is calculated from the date of surgery to the date of death or last follow-up. The temporalis tertiles represented three mutually exclusive cohorts. Binary outcomes were compared using a chi-squared (χ2) test. Means of continuous outcomes of the tertiles were first analyzed with Bartlett’s test for equal variances. If the variance ratios were not statistically significantly different, then the continuous outcomes were compared with an analysis of variance (ANOVA). If the variance ratios were statistically significantly different, then the non-parametric data were compared with a Kruskal-Wallis test. Means of continuous outcomes between two groups were first compared with a variance ratio test, followed by a Student’s t-test for equal variances or a non-parametric Welch’s t-test for unequal variances. Median/ordinal variables of the tertiles were compared with a nonparametric equality-of-medians test. Cox proportional hazards regression analyses provided an estimate of the hazard ratios (HRs) of death. Unadjusted HR tested for the likelihood of mortality with any given variable of interest. Multivariate analysis of adjusted HR controlled for clinically relevant predictors of death, along with statistically significant predictors in the univariate analysis. Kaplan-Meier estimates of time to death were categorized by average temporal tertile. The log-rank test was used to assess differences among the three Kaplan-Meier curves (i.e., tertiles). All testing was done at a statistical significance level of 0.05. All statistical analyses were performed using STATA (version 13.0, College Station, TX, USA).

## Results

Patient demographics and oncologic characteristics

We included 257 newly diagnosed GBM patients who received care at our center (Table 1). Within this cohort, 60% were males and 90.2% were Caucasian. Patients were divided into tertiles based on temporalis thickness, with tertile 1 being the thinnest and thus the most sarcopenic. We noticed statistically significant differences in demographics between tertiles; the mean age and history of CAD were both associated with smaller muscle size (p < 0.001 and p = 0.007, respectively). Patients in lower temporalis thickness tertiles had significantly lower body mass index (BMI) (p = 0.004). However, despite this association, the temporalis muscle thickness remained independently predictive of 30-day, 90-day, and overall mortality after adjustment for BMI and other demographic, clinical, and oncologic variables, indicating that temporalis thickness provides prognostic information beyond the global body mass alone. We observed no significant differences in gender, DM, HTN, seizures, HLD, stroke/TIA, COPD/asthma, chronic kidney disease (CKD), anxiety/depression, DVT/pulmonary embolism (PE), tobacco usage, and alcohol usage. We observed no statistically significant differences between tertiles related to tumor volume, focality, laterality, eloquence, and extent of resection (gross total resection vs. subtotal/biopsy) (Table 2). Patients with evidence of sarcopenia were less likely to undergo radiation with concurrent temozolomide [2] and were also more likely to die at 30 and 90 days after their surgery (p < 0.001 for all endpoints).

**Table 1:** Patient demographic information To analyze the association of temporalis size with survival, measurements of the temporalis muscle were divided into tertiles. Binary outcomes were compared using a chi-squared (χ2) test. Means of continuous outcomes were first compared with a variance ratio test, followed by a Student’s t-test for equal variances or a non-parametric Welch’s t-test for unequal variances. * Significance was assessed at P < 0.05.

	Temporalis tertile 1 % (N = 88)	Temporalis tertile 2 % (N = 84)	Temporalis tertile 3 % (N = 85)	P-value
Sex, male	60.2% (53)	60.7% (51)	60.0% (51)	0.995
Mean age ± standard deviation	72.0 ± 11.4	63.6 ± 11.5	60.1 ± 12.4	*<0.001
Race				
Caucasian	94.3% (83)	91.6% (77)	84.7% (72)	0.299
African-American	3.4% (3)	4.7% (4)	8.2% (7)	
Other	2.2% (2)	3.5% (3)	7.0% (6)	
Mean body mass index ± standard deviation	27.2 ± 5.5	26.4 ± 5.5	29.5 ± 7.6	*0.004
Diabetes mellitus	11.3% (10)	15.4% (13)	14.1% (12)	0.724
Hypertension	52.2% (46)	41.6% (35)	45.8% (39)	0.372
History of seizures	17.0% (15)	20.2% (17)	28.2% (24)	0.187
Hyperlipidemia	29.5% (26)	29.7% (25)	24.7% (21)	0.708
History of myocardial infarction/coronary artery disease	17.0% (15)	7.1% (6)	3.5% (3)	*0.007
Stroke/transient ischemic attack	7.9% (7)	5.9% (5)	1.1% (1)	0.114
Chronic obstructive pulmonary disease/asthma	10.2% (9)	4.7% (4)	9.4% (8)	0.373
Chronic kidney disease	1.1% (1)	3.5% (3)	1.1% (1)	0.421
Anxiety/depression	27.2% (24)	17.8% (15)	20.0% (17)	0.290
Deep vein thrombosis/pulmonary embolism	12.5% (11)	8.3% (7)	11.7% (10)	0.648
Tobacco use				
Never used	52.2% (46)	55.9% (47)	62.3% (53)	0.461
Current user	5.6% (5)	9.5% (8)	8.2% (7)	
Former user	42.0% (37)	34.5% (29)	29.4% (25)	
History of alcohol use	10.2% (9)	4.7% (4)	2.3% (2)	0.077

**Table 2:** Oncologic information To analyze the association of temporalis size with survival, measurements of the temporalis muscle were divided into tertiles. Binary outcomes were compared using a chi-squared (χ2) test. Means of continuous outcomes were first compared with a variance ratio test followed by student’s t-test for equal variances or a non-parametric Welch’s t-test for unequal variances. * Significance was assessed at P < 0.05.

	Temporalis tertile 1 % (N = 88)	Temporalis tertile 2 % (N = 84)	Temporalis tertile 3 % (N = 85)	P-value
Mean tumor volume (cm^3^) ± standard deviation	37.1 ± 38.7	38.6 ± 31.2	37.8 ± 31.7	0.709
Focality				
Unifocal	84.0% (74)	83.3% (70)	91.7% (78)	0.207
Multifocal	15.9% (14)	16.6% (14)	8.2% (7)	
Laterality				
Unilateral	86.3% (76)	92.8% (78)	92.9% (79)	0.232
Bilateral	13.6% (12)	7.1% (6)	7.0% (6)	
Eloquent surgical site	72.7% (64)	65.4% (55)	60.0% (51)	0.207
Resection				
Gross total resection	82.9% (73)	82.1% (69)	75.2% (64)	0.386
Subtotal resection or biopsy	17.0% (15)	17.8% (15)	24.7% (21)	
No temozolomide (TMZ) / external beam radiation therapy (EBRT)	45.4% (40)	22.6% (19)	11.7% (10)	*<0.001
30-day mortality	11.3% (10)	1.1% (1)	0.0% (0)	*<0.001
90-day mortality	28.4% (25)	8.3% (7)	1.1% (1)	*<0.001

30-day mortality

Univariate analysis identified patient age as a unique factor associated with mortality within 30 days of initial surgery for GBM (OR 2.45, p = 0.003) (Table 3). Univariate analysis also identified temporalis thickness as significantly associated with 30-day mortality (OR 0.08, p = 0.010). When adjusting for potentially confounding variables in multivariate analysis, age no longer became significantly associated with 30-day mortality (p = 0.102), but temporalis thickness remained significant (OR 0.10, 95% CI 0.01-0.79, p = 0.030). Clinically speaking, an OR of 0.10 for temporalis thickness implies that the odds of death decreased by 90% for every 1-mm increase in thickness. Analysis demonstrated that mortality at 30 days had no association with gender, past medical history, tumor focality, tumor volume, tumor eloquence, or extent of resection.

**Table 3:** 30-day mortality Measurements of the temporalis muscle were divided into tertiles. Logistic regression with unadjusted odds ratio (univariable regression) and adjusted odds ratio (multivariable regression) for 30-day mortality following surgery. The odds ratio (OR) of 0.10 for temporalis thickness means that the odds of death decreased by 90% for every 1-mm increase in thickness. All regressions were adjusted for age, sex, race, mean body mass index, and medical co-morbidities (e.g., diabetes mellitus, hypertension, chronic obstructive pulmonary disease (COPD), etc.). * Significance was assessed at P < 0.05.

	Unadjusted odds ratio (95% confidence interval)	P-value	Adjusted odds ratio (95% confidence interval)	P Value
Demographics				
Sex, male	3.08 (0.65–14.56)	0.155	5.72 (0.76–42.85)	0.090
Decade of life	2.45 (1.34–4.47)	*0.003	1.88 (0.88–4.00)	0.102
Hypertension	1.38 (0.41–4.67)	0.595	0.87 (0.17–4.38)	0.867
Myocardial infarction/coronary artery disease	2.26 (0.45–11.13)	0.315	1.12 (0.15–8.04)	0.910
Chronic kidney disease	6.05 (0.61–59.19)	0.122	24.63 (0.51–1174.77)	0.104
Tobacco use	1.15 (0.61–2.17)	0.657	1.04 (0.47–2.33)	0.905
Anxiety/depression	0.79 (0.16–3.76)	0.767	0.74 (0.11–5.07)	0.766
Seizures	0.79 (0.16–3.76)	0.767	2.04 (0.23–17.57)	0.514
Diabetes mellitus	0.62 (0.07–5.02)	0.657	0.40 (0.03–4.24)	0.448
Chronic obstructive pulmonary disease/asthma	2.65 (0.53–13.17)	0.232	1.87 (0.21–16.22)	0.570
Oncologic factors				
Focality, multifocal	1.43 (0.29–6.93)	0.654	1.68 (0.19–14.59)	0.635
Tumor volume	1.00 (0.98–1.01)	0.906	1.00 (0.98–1.02)	0.566
Eloquence	2.37 (0.50–11.24)	0.275	1.43 (0.18–10.95)	0.730
Laterality, bilateral	2.26 (0.45–11.13)	0.315	2.62 (0.24–27.70)	0.422
Treatments				
Resection, subtotal resection/biopsy	0.39 (0.04–3.13)	0.377	0.43 (0.03–5.54)	0.522
Sarcopenia				
Mean temporalis thickness	0.08 (0.01–0.55)	*0.010	0.10 (0.01–0.79)	*0.030

90-day mortality

On univariate analysis, age was again associated with mortality at 90 days (OR 2.01, P < 0.001), as was the history of CAD (OR 4.16, P = 0.003), eloquent tumor (OR 2.54, P = 0.003), and bilateral tumors (OR 4.16, P = 0.003) (Table 4). Multivariate analysis revealed bilateral tumors to be the only demographic factor that was significantly associated with 90-day mortality (OR 4.75, 95% CI 1.14-19.69, P = 0.032). In addition, sarcopenia was also significantly associated with mortality at this time point; patients in Tertile 1 had a 3.5-time and 25-time higher likelihood of mortality at 90 days as compared to Tertiles 2 (OR 3.52, P = 0.021) and 3 (OR 24.68, P = 0.003).

**Table 4:** 90-day mortality Measurements of the temporalis muscle were divided into tertiles. Logistic regression with unadjusted odds ratio (univariable regression) and adjusted odds ratio (multivariable regression) for 90-day mortality following surgery. Mean temporalis size is included; tumor markers are excluded. All regressions were adjusted for age, sex, race, mean body mass index, and medical co-morbidities (e.g., diabetes mellitus, hypertension, chronic obstructive pulmonary disease (COPD), etc.). * Significance was assessed at P < 0.05.

	Unadjusted odds ratio (95% confidence interval)	P	Adjusted odds ratio (95% confidence interval)	P
Demographics				
Sex, male	0.76 (0.36–1.59)	0.469	0.70 (0.27–1.79)	0.465
Decade of life	2.01 (1.42–2.85)	*<0.001	1.52 (0.97–2.37)	0.062
Hypertension	1.43 (0.68–2.99)	0.335	1.18 (0.43–3.23)	0.744
Myocardial infarction/coronary artery disease	4.16 (1.61–10.69)	*0.003	2.61 (0.79–8.61)	0.115
Chronic kidney disease	4.75 (0.76–29.57)	0.095	9.81 (0.90–106.29)	0.060
Tobacco use	1.26 (0.86–1.85)	0.230	1.32 (0.81–2.15)	0.265
Anxiety/depression	0.77 (0.30–1.97)	0.592	0.43 (0.13–1.36)	0.152
Seizures	0.77 (0.30–1.97)	0.592	2.60 (0.74–9.13)	0.134
Diabetes mellitus	1.88 (0.74–4.74)	0.179	1.67 (0.52–5.31)	0.382
Chronic obstructive pulmonary disease/asthma	2.32 (0.78–6.82)	0.126	1.10 (0.25–4.80)	0.891
Oncologic factors				
Focality, multifocal	1.49 (0.56–3.92)	0.416	0.74 (0.19–2.88)	0.665
Tumor volume	1.00 (0.98–1.01)	0.972	1.00 (0.99–1.01)	0.575
Eloquence	2.54 (1.01–6.43)	*0.048	2.16 (0.65–7.16)	0.206
Laterality, bilateral	4.16 (1.61–10.69)	*0.003	4.75 (1.14–19.69)	*0.032
Treatments				
Resection, subtotal resection/biopsy	0.36 (0.10–1.25)	0.110	0.43 (0.09–1.88)	0.265
Sarcopenia				
Temporalis size thickness				
Tertile 1 vs. Tertile 2	4.36 (1.77–10.75)	*0.001	3.52 (1.20–10.29)	*0.021
Tertile 1 vs. Tertile 3	33.33 (4.39–252.61)	0.060	24.68 (2.90–209.88)	*0.003
Tertile 2 vs. Tertile 3	7.63 (0.91–63.49)	0.060	7.05 (0.77–63.36)	0.083

Overall mortality

When looking at overall mortality from the initial surgery (Table 5), several clinical and demographic factors were associated with mortality on both univariate and multivariate analysis, including age (OR 1.28, p < 0.001 on univariate; OR 1.30, 95% CI 1.11 - 1.52, p = 0.001 on multivariate), seizures (OR 0.72, p = 0.047 on univariate; OR 0.61, 95% CI 0.39-0.94, p = 0.025 on multivariate), and DM (OR 1.81, p = 0.001 on univariate; OR 1.66, 95% CI 1.04-2.65, p = 0.031 on multivariate). Oncologic factors associated with mortality on both univariate and multivariate analysis include multifocal tumor (OR 2.12, p < 0.001 on univariate; OR 2.02, 95% CI 1.08-3.76, p = 0.026 on multivariate), eloquent cortex involvement (OR 1.72, p < 0.001 on univariate; OR 1.50, 95% CI 1.03-2.18, p = 0.032 on multivariate), subtotal resection or biopsy (OR 0.55, p < 0.001 on univariate; OR 0.54, 95% CI 0.35-0.84, p = 0.007 on multivariate), radiation with chemotherapy (OR 2.41, p < 0.001 on univariate; OR 3.59, 95% CI 2.41-5.36, p < 0.001 on multivariate), and MGMT methylation status (OR 0.49, p < 0.001 on univariate; OR 0.44, 95% CI 0.31-0.63, p < 0.001 on multivariate). IDH mutation status (OR 0.33, p = 0.017) and laterality (OR 2.43, p < 0.001) lost significance on multivariate analysis. Although several demographic, clinical, oncologic, and treatment-related variables were associated with mortality, temporalis muscle thickness remained a statistically significant predictor of survival on multivariate analysis after adjustment for these factors, confirming sarcopenia as an independent predictor of mortality.

**Table 5:** Overall mortality Measurements of the temporalis muscle were divided into tertiles. Logistic regression with unadjusted hazard ratio (univariable regression) and adjusted hazard ratio (multivariable regression) for overall mortality following surgery. Mean temporalis size and tumor markers are included. All regressions were adjusted for age, sex, race, mean body mass index, and medical co-morbidities (e.g. diabetes mellitus, hypertension, COPD, etc.). * Significance was assessed at P < 0.05.

	Unadjusted odds ratio (95% confidence interval)	P-value	Adjusted odds ratio (95% confidence interval)	P Value
Demographics				
Sex, male	1.07 (0.82–1.39)	0.584	1.18 (0.82–1.69)	0.355
Decade of life	1.28 (1.16–1.42)	<0.001	1.30 (1.11–1.52)	*0.001
Hypertension	1.24 (0.96–1.61)	0.090	1.06 (0.74–1.53)	0.723
Myocardial infarction/coronary artery disease	1.44 (0.93–2.24)	0.099	1.07 (0.63–1.83)	0.781
Chronic kidney disease	0.69 (0.25–1.87)	0.468	0.74 (0.21–2.65)	0.653
Tobacco use	1.05 (0.92–1.21)	0.426	1.17 (0.97–1.40)	0.088
Anxiety/depression	0.93 (0.68–1.27)	0.663	0.86 (0.57–1.27)	0.458
Seizures	0.72 (0.53–0.99)	0.047	0.61 (0.39–0.94)	*0.025
Diabetes mellitus	1.81 (1.25–2.61)	0.001	1.66 (1.04–2.65)	*0.031
Chronic obstructive pulmonary disease/asthma	0.84 (0.52–1.36)	0.499	0.89 (0.47–1.68)	0.742
Oncologic factors				
Focality, multifocal	2.12 (1.46–3.06)	<0.001	2.02 (1.08–3.76)	*0.026
Tumor volume	0.99 (0.99–1.00)	0.769	1.00 (1.00–1.01)	0.029
Eloquence	1.72 (1.30–2.26)	<0.001	1.50 (1.03–2.18)	*0.032
Laterality, bilateral	2.43 (1.59–3.73)	<0.001	1.30 (0.62–2.73)	*0.480
Treatments				
Resection, subtotal resection/biopsy	0.55 (0.39–0.77)	<0.001	0.54 (0.35–0.84)	*0.007
TMZ / EBRT	2.41 (1.81–3.20)	<0.001	3.59 (2.41–5.36)	*<0.001
Sarcopenia				
Temporalis size thickness				
Tertile 1 vs. Tertile 2	2.42 (1.76–3.31)	<0.001	2.43 (1.61–3.67)	*<0.001
Tertile 1 vs. Tertile 3	3.73 (2.70–5.15)	<0.001	2.43 (1.53–3.84)	*<0.001
Tertile 2 vs. Tertile 3	1.54 (1.12–2.12)	0.008	1.00 (0.65–1.52)	*0.998
Molecular markers				
MGMT (tumor marker)	0.49 (0.36–0.67)	<0.001	0.44 (0.31–0.63)	*<0.001
IDH (tumor marker)	0.33 (0.13–0.82)	0.017	0.90 (0.30–2.67)	*0.857

**Figure 2:**
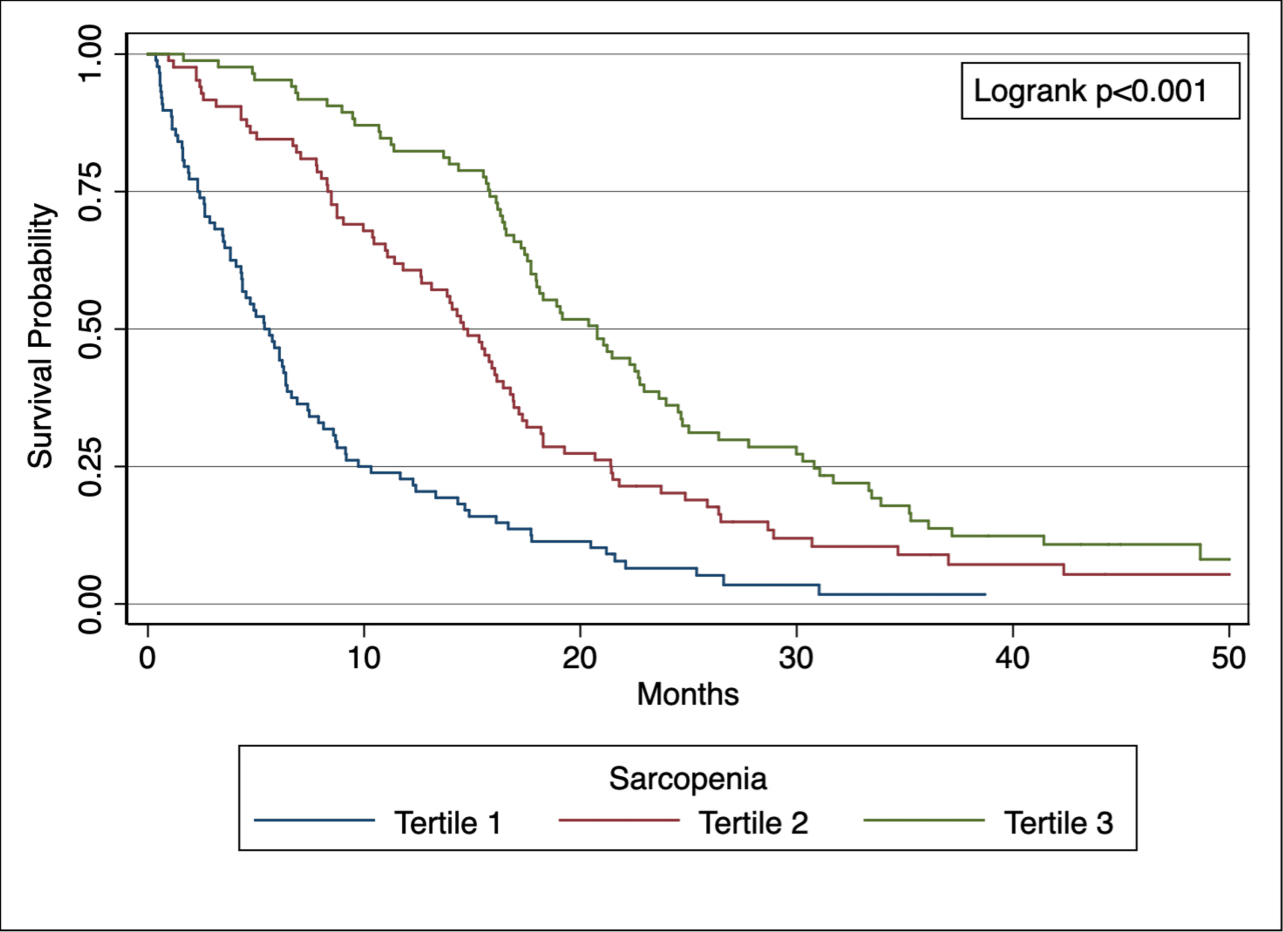
Kaplan-Meier curves represent differences in survival over time (months) among the tertiles of temporalis muscle thickness (log-rank p < 0.001). Measurements of the temporalis muscle were divided into tertiles.

When comparing the adjusted odds of overall mortality based on temporalis size, sarcopenia was again significantly associated with death; patients in Tertile 1 had a 2.43-fold increased likelihood of mortality at 90 days as compared to Tertiles 2 (OR 2.43, p < 0.001) and 3 (OR 2.43, p < 0.001). Kaplan-Meier survival curves visually represent differences in mortality (Fig. 2, log-rank p < 0.001).

## Discussion

Key results

In this retrospective cohort study of patients with newly diagnosed GBM, we identified that sarcopenia, as measured by temporalis thickness, is a strong predictor of 30-day, 90-day, and overall mortality. Patients who are more sarcopenic are more likely to die at these time-points. Sarcopenia at the time of surgery also predicted survival independent of patient demographics (age, gender, etc.), past medical and social history, tumor factors (size, eloquence, laterality), treatment factors (extent of resection, radiation, and chemotherapy), and common tumor markers (MGMT and IDH status). The survival disparity for patients with and without sarcopenia was most noticeable at 90 days, with sarcopenia being associated with about a 25x greater odds of death at this time point. Sarcopenia also compares favorably with other frequently used prognostic factors. Patients in the smallest temporalis thickness, tertile 1, had an odds ratio of overall survival of 0.41 versus the largest tertile, whose impact is comparable to the improvement seen by adding radiation treatment with concurrent temozolomide (OR 0.27) [2], gross total resection (OR 0.54) [3], MGMT methylation status (OR 0.44) [7], and IDH mutation status (OR 0.33) [8].

The strong association between temporalis muscle thickness and early (30-day, 90-day) as well as overall mortality highlights the critical role of baseline physiologic reserve in determining outcomes for patients with newly diagnosed glioblastoma. While glioblastoma prognosis has traditionally been driven by tumor-specific factors and treatment variables, our findings emphasize that overall patient health status, specifically sarcopenia and frailty, also represent important determinants of survival.

Sarcopenia reflects decreased muscle mass and is increasingly recognized as a surrogate for frailty, impaired immune function, metabolic dysregulation, and reduced tolerance to physiologic stress [16-18]. In oncology populations, sarcopenia has been associated with increased postoperative complications, decreased tolerance of chemotherapy and radiation, and shortened survival [19-25]. Our results extend these observations to glioblastoma, demonstrating that sarcopenia assessed through temporalis muscle thickness provides meaningful prognostic information independent of age, comorbidities, tumor characteristics, molecular markers, and treatment factors. Temporalis muscle thickness performed similarly to established prognostic factors such as extent of resection, MGMT methylation status, IDH mutation status, and receipt of postoperative chemoradiation. The strength of its association with mortality suggests that frailty may be as important as traditional tumor-based markers in predicting survival. These findings support a more comprehensive approach to glioblastoma care that considers both tumor characteristics and overall patient health when counseling patients and selecting treatment strategies. From a clinical standpoint, temporalis muscle thickness offers several advantages as a biomarker. It can be rapidly measured on standard perioperative MRI without additional cost, radiation exposure, or specialized testing. This makes it highly practical for routine clinical use, especially in settings where advanced molecular profiling may be unavailable. Incorporating temporalis thickness into preoperative assessment may help identify patients who are unlikely to benefit from aggressive surgical or adjuvant therapies and who may instead be better served by more conservative or palliative approaches. Conversely, patients with preserved muscle mass, even at advanced age, may be appropriate candidates for more aggressive intervention.

Although BMI differed across temporalis thickness tertiles, BMI and temporalis muscle thickness represent distinct physiological constructs. BMI reflects total body mass and does not differentiate lean muscle from adipose tissue, whereas temporalis thickness directly measures skeletal muscle and therefore more accurately reflects sarcopenia and frailty [16-18]. Prior studies in oncology populations have demonstrated that sarcopenia may be present even in patients with normal or elevated BMI (“sarcopenic obesity”) and is associated with worse clinical outcomes [19-25]. Consistent with this concept, temporalis thickness remained independently associated with mortality after adjustment for BMI, supporting its role as a complementary and superior marker of frailty. These findings suggest that BMI and temporalis thickness can be used together clinically, with temporalis thickness offering additional prognostic value beyond traditional metrics.

Future work should focus on establishing standardized temporalis thickness cutoff values, evaluating inter-rater reliability across institutions, and integrating sarcopenia metrics into existing prognostic models. Prospective validation is also warranted to determine whether temporalis thickness can predict treatment tolerance, postoperative morbidity, and quality-of-life outcomes. Ultimately, incorporation of frailty assessment into glioblastoma care may allow for more personalized, patient-centered treatment strategies.

Interpretation, generalizability, and limitations

This study introduces sarcopenia (a marker of patient frailty) as an accurate, simple, and independent biomarker of short-term (30 and 90 days) and long-term (overall) survival in patients with GBM. Sarcopenia as measured by temporalis thickness is a quick and easy measurement, performed on a standard peri-operative MRI, and does not require specialized labs or testing while providing an objective assessment of patient frailty and survival.

From a practical perspective, sarcopenia may be used in the preoperative setting to assist with risk stratification and individualized treatment planning rather than to replace established standards of care. While gross total resection followed by chemoradiation remains the standard treatment paradigm for eligible patients, temporalis muscle thickness may provide additional prognostic context to guide shared decision-making. In cases where patients present with advanced age but are clinically robust, or conversely are younger but physically deconditioned, temporalis muscle measurements offer an objective assessment of overall health status that may help inform surgical and therapeutic decisions. Incorporating sarcopenia assessment may help identify patients at higher risk for early mortality and treatment-related morbidity, allowing clinicians to tailor treatment intensity, optimize supportive care, and consider earlier integration of palliative services when appropriate. Conversely, patients with preserved muscle mass, even at advanced age, may be more likely to tolerate aggressive surgical and adjuvant therapies. Regardless of age, comorbidities, tumor characteristics, or planned postoperative therapy, sarcopenia provides insight into overall patient resilience and survival and may therefore help inform the selection of appropriate candidates for specific interventions.

We expect our results to be widely generalizable; our data is derived from a large prospective tumor bank from a specialized tertiary care center, and therefore may be applied to a wide range of geographies. We are limited by the nature of this study, which is a single-center retrospective cohort of a prospective database. There may be hidden biases that we are unable to account for due to incomplete or inaccurate medical records. A clear example in this study is that only 10 patients had a documented IDH mutation in our cohort. Subsequently, IDH mutation was not predictive of overall survival in our study (Table 5), in contrast to the established literature (Type II error) [29]. However, the prevalence of IDH mutation in GBM is low (~7.13%) [8], and so a larger and more focused study is required to compare the efficacy of frailty/sarcopenia versus IDH status to predict survival. We are also limited by the demographics of our patients (located primarily in our state), with about 90% being Caucasian.

Multiple areas of future research are required. The reliability of temporalis measurements needs to be definitively established before it can be applied across multiple centers, which requires a dedicated reliability study. Our results also require prospective validation in another dataset and further investigation on whether this biomarker contributes prognostic information to existing clinical models. Whether temporalis thickness can predict post-operative morbidity after craniotomy also needs to be established. Further research is necessary to create definitive temporalis thickness cutoff points that can be translated to clinical practice for guidelines and recommendations.

With the growing role of artificial intelligence (AI) in neurosurgery, objective and readily available biomarkers such as temporalis muscle thickness may support future predictive models aimed at selecting appropriate patients for specific interventions. Creating these models requires large datasets that incorporate both patient and tumor-specific factors, consistent with current AI efforts in neurosurgery [31].

## Conclusions

Temporalis thickness at the time of GBM diagnosis (a surrogate for frailty/sarcopenia) accurately and strongly predicts 30-day, 90-day, and overall mortality. Our results were independent of patient demographics, past medical history, tumor factors (size, eloquence, laterality), treatment factors (extent of resection, post-surgical treatment), and common tumor markers (MGMT and IDH status). The ability of temporalis thickness to predict overall survival was comparable to other recognized factors, including treatment with postoperative radiation and temozolomide, gross total resection, MGMT methylation status, and IDH mutation status.
